# Incorporation of T-cell epitopes from tetanus and diphtheria toxoids into in-silico-designed hypoallergenic vaccine may enhance the protective immune response against allergens

**DOI:** 10.22038/ijbms.2020.39749.9422

**Published:** 2020-05

**Authors:** Ali Ghasemi, Reza Falak, Mohsen Mohammadi, Soheila June Maleki, Mohamad-Ali Assarezadegan, Mojtaba Jafary

**Affiliations:** 1 Department of Biochemistry and Hematology, Faculty of Medicine Semnan University of Medical Sciences, Semnan, Iran; 2 Immunology Research Center, Institute of Immunology and Infectious Diseases, Iran University of Medical Sciences, Tehran, Iran; 3 The Persian Gulf Marine Biotechnology Research Center, the Persian Gulf Biomedical Sciences Research Institute, Bushehr University of Medical Sciences, Bushehr, Iran; 4 US Department of Agriculture, Agricultural Research Service, Southern Regional Research Center, New Orleans, Louisiana, USA

**Keywords:** Allergen, Chenopodium album, Diphtheria-tetanus vaccine, Epitope, T lymphocyte

## Abstract

**Objective(s):**

New generation of allergy vaccines is capable of promoting the development of protective IgG and blocking the functionality of allergen-specific IgE. We incorporated universal and powerful T-cell epitopes from tetanus and diphtheria toxoids (TD epitope) into recombinant Che a 2, the well-known allergic profilin of *Chenopodium* *album*, to determine its immunological properties.

**Materials and Methods:**

The sequence and accordingly the structure of the recombinant Che a 2 was altered to generate a hypoallergenic variant (rChe a 2.rs). Moreover, TD epitope was incorporated to produce a novel vaccine that was nominated as rChe a 2.rs^T.D^. The effect of treatment with these variants was evaluated on the generation of allergen-specific IgG class, as well as lymphocyte proliferation in mice. Moreover, IgE-binding characteristics of the allergic patients’ sera were determined by ELISA and proliferation and cytokine production was measured in T-cells.

**Results:**

ELISA and dot blot revealed strong reduction of the IgE-reactivity of human sera to the variants of Che a 2 as compared to the wild-type molecule. Furthermore, Che a 2.rs and Che a 2.rs^T.D^ induced much lower levels of IL5 and IL13 secretion from allergic patients’ PBMCs in comparison to wild-type Che a 2 protein. In mice, rChe a 2.rs^T.D^ induced high titers of Che a 2-specific IgG antibody capable of blocking IgE-binding to rChe a 2 and induced lymphocyte proliferation more potently than rChe a 2.rs.

**Conclusion:**

Collectively, incorporation of T-cell epitopes of tetanus and diphtheria into hypoallergenic vaccines can dramatically enhance anti-allergic immune mechanisms, particularly in poor responders.

## Introduction

Allergy, particularly respiratory allergy, is the most common health issue worldwide, and its incidence is increasing in the developing and industrial countries (1-3). Type I allergy is caused by allergic proteins that can cross-link FceRI-bound IgE antibodies on effector cells such as mast cells and basophils, and subsequently lead to releasing of the mediators, including histamine and leukotrienes (4). Availability of at least two epitopes on the surface of the allergen is crucial for successful cross-linking of the membranous FceRI molecules of the effector cells. These IgE epitopes can be linear or conformational. Linear epitopes contain continuous amino acids, while conformational epitopes are composed of discontinuous amino acids brought together in the three-dimensional structure of the protein (5). The allergenic characteristics of the inhaled and food allergens mostly depend on their conformation; therefore, destruction of the native folding or conformation of the allergens will strongly reduce their IgE-reactivity (5, 6). Allergen-specific immunotherapy (SIT), the only available treatment for allergic disease, involves the administration of increasing doses of the purified allergen, allergen extracts, or whole food to induce desensitization. An effective mechanism of desensitization is development of allergen-specific protective IgG antibodies (7, 8). However, sometimes low protein content and variable quality of the crude extracts complicate the clinical application of these extracts in SIT (9-12). 

The use of purified allergens or hypoallergenic variants of the allergens for SIT removes or reduces the problems regarding the content and quality of an allergen in crude extract and may also reduce the possible side effects during the treatment (13, 14). Several strategies may be employed to improve these benefits and reduce the possible side effects. One strategy involves the incorporation of T-cell-specific epitopes in order to induce tolerance in allergen-specific T-cells (15, 16). A novel approach is to genetically alter the amino acid sequence of the allergen by making mutations, deletion, or inversion in the DNA sequence to generate a hypoallergenic variant (17-19). Manipulation of the allergen to generate hypoallergenic vaccine may remove CD4^+^ T cell epitopes that are essential for T-cell-dependent antibody responses. In this case, the hypoallergen may induce production of allergen-specific IgG or other T cell-dependent protective immunological responses. Therefore, the concern for the abolishment of T-cell epitopes complicates vaccine design via genetic engineering strategies (19, 20).

The current study presents a strategy to enhance the potency of the recombinant-hypoallergenic vaccines by incorporating two strong and universal T helper cell (Th) epitopes from tetanus (T 830-844) and diphtheria toxoid (D 331-345) (21, 22) linked to the internal cathepsin cleavage site, in order to further enhance the activation and response of the T cells. We believe that this approach will alleviate concerns and limitations related to the potential loss of allergen-specific T-cell epitopes in genetically engineered vaccines. 

In the current study, a genetically modified hypoallergenic variant of the profilin from *Chenopodium album* (Che a 2), which is a highly cross-reactive and clinically relevant pan-allergen in pollens of plants, weeds, and grasses (23-25), was generated according to the tail-to-head exchanging strategy. Following the recombination of the N-terminal and C-terminal fragments of the allergen, as described by Westritschnig *et al.* (26), TD epitopes were added on the C-terminal end of the protein. The reconstructed allergen was then used to test for IgE-binding and to treat peripheral blood mononuclear cells from allergic patients. Our study demonstrates that the T cells recognize TD epitopes incorporated into hypoallergenic Che a 2.rs (Che a 2.rs^T.D^), which may lead to altering the immune response and boosting IgG production against profilin.

## Materials and Methods

***Molecular modeling of the Che a 2 allergen and designing hypoallergenic Che a 2 containing T.D epitope***

**Figure 1:**
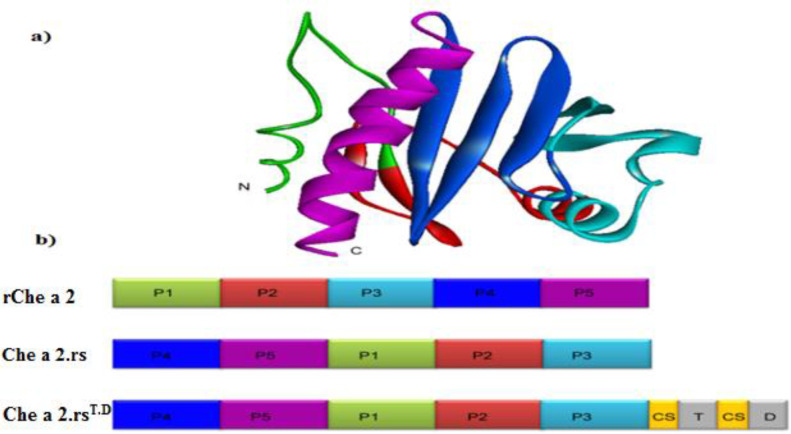
Ribbon diagram and linear depiction of Che a 2, Che a 2.rs, and Che a 2.rs^T.D^

The ribbon structure of Che a 2 (Genbank accession No: AY082337.1) was constructed by a fully automated protein structure homology-modeling server. The profilin from *Hevea brasiliensis* (27), Birch (28), and *Arabidopsis thaliana* (29) was employed to generate a model for profilin from *C. album*, Che a 2. The quality of the constructed model was assessed by the QMEAN server.Considering previously identified IgE epitopes on profilin (26), Che a 2-encoding cDNA was reconstructed through tail-to-head recombination of the P1 peptide at N-terminal and P5 at C-terminal peptide of Che a 2 to generate a hypoallergenic variant which was termed as Che a 2.rs. Moreover, T-cell epitopes including TD epitopes with cathepsin cleavage sites were added to the C-terminal end of Che a 2.rs, resulting in Che a 2.rs^T.D^ construct (Figure 1).

***Expression and purification of r.Che a 2.rs and r.Che a 2.rs*** ^T.D^

Che a 2.rs (399 bp) and Che a 2.rs^T.D ^(507 bp) cDNAs (codons optimized for expression in *Escherichia coli*) were chemically synthesized (Pepmic Co, China), and inserted into the Nde1/XhoI sites of pET21-a (Novagen, USA) so that the 6-His-tagged region located at C-terminus. The inserted nucleotide sequence was confirmed by restriction enzyme digestion and by means of automated DNA sequencing. Then *E. coli* BL21 cells (Novagen, USA) were transformed by the construct and selected on Luria-Bertani (LB) plates containing 100 µg/ml of ampicillin. A typical colony was picked and after overnight culture at 37 ^°^C, inoculated into 300 ml LB medium containing 100 µg/ml of ampicillin and incubated at 37 ^°^C until the absorbance reached 0.5 at 600 nm. Then, the expression of the recombinant proteins was induced by adding 1 mM isopropyl-thiogalactopyranoside (IPTG) for approximately four hours. Finally, bacterial cells were harvested by centrifugation at 4000 rpm for 10 min, and the pellet was re-suspended in 5 ml of lysis buffer (100 mM/l NaH_2_PO_4_; pH 7.0, 10 mM Tris-Cl, 150 mM NaCl), and the bacterial cells were disrupted by ultrasonication five times at 22 kHz at 20-sec intervals on ice. Both proteins were expressed as insoluble inclusion bodies and existed in the pellet fraction. The pellets were solubilized (6 M urea, 100 mM NaH_2_PO_4_, 10 mM Tris-Cl; pH 8) for two hours at 37 ^°^C. Insoluble proteins were removed by centrifugation (10000 rpm for 15 min at 4 ^°^C) and the urea concentration was reduced to 2 mM with stepwise dialysis in order to allow refolding of the protein. The 6-His-tagged recombinant proteins were purified by nickel affinity chromatography (Ni-NTA agarose) (Thermo Fisher Scientific, USA) according to the manufacturer’s instructions, and dialyzed against 50 mM NaH_2_PO_4_, pH 7. Purified rChe a 2 protein (30) containing Trx-tag was kindly gifted by Dr. A. Varasteh (Bu-Ali Research Center, Mashhad, Iran), and its Trx-tag was removed. 

***Allergic patients***

Sera from 15 *C. album*-allergic patients (9 males and 6 females) with the mean age of 30.13 years were utilized to characterize the designed hypoallergenic molecules (Table 1). The mean specific IgE of the patients was 7.74±1.29 IU/ml for *C. album* as determined by enzyme allegro sorbent test (EAST) and all of them had a clinical history of allergy and positive skin prick test for *C. album*. The obtained serum samples were investigated for the level of Che a 2-specific IgE (31) and recombinant Che a 2 (rChe a 2) protein. Serum samples from non-allergenic individuals (n:7) were used as control. The Ethics Committee of the Persian Gulf Biomedical Sciences Research Institute approved the study methods, and all participants signed the informed consent forms before starting the project.

***Evaluation of IgE-binding capacity of recombinant r.Che a 2, r.Che a 2-rs., and r.Che a 2-rs*** ^T.D^ ***with indirect ELISA and dot blot***

IgE-reactivity of recombinant proteins, r.Che a 2, r.Che a 2-rs, and r.Che a 2-rs^T.D^ was determined by an in-house developed indirect ELISA and dot blot assay. Indirect ELISA was performed as previously described (32). Briefly, recombinant proteins (2 μg/well) were coated into the wells and then the wells were blocked with 2% bovine serum albumin (BSA, Sigma, MO, USA). Wells were exposed to 1:5 diluted sera of patients with allergy to rChe a 2 (n:15) or non-allergenic individuals’ sera (n:7) and incubated with 1:2000 diluted biotin-labeled anti-human IgE (Abcam, UK). Wells were incubated for one hour with 1:30000 diluted HRP-conjugated streptavidin (Sigma, MO, USA). After addition of TMB/H_2_O_2_ chromogen/substrate (Sigma, MO, USA), the plates were incubated in the dark for 15 min. The reaction was stopped by addition of 100 μl of 2 N H_2_SO_4_. Finally, the optical density (OD) of the wells was measured at 450/630 nm. 

Dot blot was performed as previously described (33). Briefly, 2 μg of recombinant proteins were coated on nitrocellulose membrane disks (5 mm diameter) (Sigma, MO, USA), blocked with 2% BSA and incubated overnight with 1/5 diluted sera of patients with allergy (n: 10). After incubation with 1:2000 diluted biotin-labeled anti-human IgE (Abcam, UK) and subsequently with 1:30000 diluted HRP conjugated streptavidin (Sigma, MO, USA) for 1 hr at room temperature, immuno-reactivity of the coated disks was revealed by a chemiluminescent substrate (Amersham, GE Healthcare Life Science) in a gel documentation system (G:BOX Chemi XX9 gel doc system, UK). 

***Immunization of mice and evaluation of IgG antibody response***

Six groups of seven-week-old female BALB/c mice (5 mice per group) were either subcutaneously pre-vaccinated with DPT vaccine or left untreated. Three weeks later, mice were immunized three times (in two-weeks intervals) with 15 μg of r.Che a 2, r.Che a 2.rs, or r.Che a 2.rs^T.D^ pre-adsorbed on aluminum hydroxide gel, and blood samples were collected from the tail vein after each immunization and two weeks after the last immunization. To investigate whether incorporation of strong T-cell epitopes in vaccines could enhance a protective antibody response under sub-optimal vaccine conditions, such as low doses of vaccine in poor responders such as aged mice (10 months old), we performed another experiment. For this purpose, 4 groups of nine-month-old female BALB/c mice (five mice per group) were either pre-vaccinated with DPT vaccine and/or left unvaccinated; and 28 days later, the two groups were immunized with 1 μg of r.Che a 2, r.Che a 2.rs, or r.Che a 2.rs^T.D^ pre-adsorbed on aluminum hydroxide. Sera were obtained 0, 7, and 14 days post-immunization via the tail vein. The production of rChe a 2-specific IgG in immunized mice was assessed by indirect ELISA. The microplate wells were coated with rChe a 2 (2 µg/well) and incubated with 1:500 diluted immunized mice sera. After the washing step, wells were probed with 1:2000 diluted HRP-conjugated goat anti-mouse IgG (Abcam, UK) for two hours at room temperature. Finally, TMB/H_2_O_2_ chromogen/substrate (Sigma, MO, USA) was added and OD of r.Che a 2-specific IgG response was measured at 450/630 nm. The corresponding pre-immunized mice sera were used as negative control in this experiment. Antibody titer was defined as half-maximal binding that is reciprocal to dilution presented at half-maximal optimal density (OD_50_).

***Competitive inhibition ELISA (ciELISA) for determination of the allergic patients’ IgE-binding capacity to r.Che a 2***

Inhibition of allergic patients’ IgE-binding capacity to rChe a 2 was determined using immunized mice sera by competitive inhibition ELISA (ciELISA). ELISA wells were coated with r.Che a 2 (1 μg/well), and incubated with serum mixtures comprising decreasing amounts of sera from immunized mice (final dilution ranging from 1:50 to 1:50000) as the competitive antibody and pooled sera (final dilution 1:5) from allergic patients (n: 10) according to the following combination:

A) No competitor: 50 µl of 1:2.5 dilution of patients pooled sera+50 µl of 1:25 dilution of non-immunized mice sera; 

B) 1:50-diluted competitor: 50 µl of 1:2.5 dilution of patients pooled sera+50 µl of 1:25 dilution of immunized mice sera; 

C) 1:100-diluted competitor: 50 µl of 1:2.5 dilution of patients pooled sera+50 µl of 1:50 dilution of immunized mice sera; 

D) 1:500-diluted competitor: 50 µl of 1:2.5 dilution of patients pooled sera+50 µl of 1:250 dilution of immunized mice sera;

E) 1:5000 diluted competitor: 50 µl of 1:2.5 dilution of patients pooled sera+50 µl of 1:2500 dilution of immunized mice sera; 

F) 1:50000 diluted competitor: 50 µl of 1:2.5 dilution of patients pooled sera+50 µl of 1:25000 dilution of immunized mice sera

After the washing steps, wells were incubated with 1:2000 diluted biotin-labeled anti-human IgE (Abcam, UK), and subsequently, with 1:30000 diluted HRP conjugated streptavidin (Sigma, MO, USA) for one hour at RT. The percentage of inhibition was calculated as follows: (OD_z _–OD_c _/ OD_z_)×100.

Where OD_z _and OD_c _represent the optical density of samples without competitor (zero competitor), and optical density of the sample with competitor, respectively. 

***Cell Proliferation assay (MTT) and cytokine response of PBMCs***

PBMCs were isolated from heparinized whole blood samples of five patients with clinical history and typical symptoms (rhinitis, conjunctivitis) for allergy to pollens who had a positive prick test for *C. album* and had specific IgE to Che a 2 (mean age:42 years) by Ficoll density gradient centrifugation. Splenocytes were stimulated with r.Che a 2, r.Che a 2.rs, or r.Che a 2.rs^T.D^, and the proliferation of the cells was assessed using Vybrant^®^ MTT Cell Proliferation Assay Kit (Thermo Scientific, USA) according to the manufacturer’s instructions. For this purpose, PBMCs were incubated in 48-well microplates in RPMI 1640 supplemented with fetal bovine serum, penicillin (100 U/ml), and streptomycin (100 μg/ml) (Sigma, MO, USA) at the density of 1×10^6^ cell/well. The cells were stimulated with r.Che a 2, r.Che a 2.rs, r.Che a 2.rs^T.D^ (10 ng/μl), or IL-2 (5 U/well, Roche Diagnostics, Rotkreuz, Switzerland) as a positive control. After seven days, lymphocyte proliferation was determined and presented as proliferation index (OD^570^ of stimulating cells/mean OD^570^ of control cells). Pro-allergenic cytokines, including IL-4, IL-5, and IL-13 were quantified in the supernatant of PBMC cultures stimulated with r.Che a 2, r.Che a 2.rs, and r.Che a 2.rs^TD ^using RayBiotech ELISA kit (RayBiotech, Inc.).

## Results

***Che a 2.rs*** ^T.D^ ***was designed according to tail-to-head recombination strategy***

The ribbon structure of Che a 2 and the restructured constructs are shown in Figure 1. In this figure, the color of the ribbon diagram in Panel A corresponds to the linear forms in panel B, depicting the changes made to native Che a 2 to produce the hypoallergenic variant (Che a 2rs), as well as the location of the incorporated T-cell epitopes and cathepsin cleavage sites in Che a 2rsTD. P1, an unordered loop at N-terminal end, and alpha-helical P5 peptides at the C-terminal end are the known IgE-binding sites in profilin (26). 

***Expression, purification, and IgE-reactivity of Che a 2, Che a 2.rs, and Che a 2.rs*** ^T.D^

**Figure 2:**
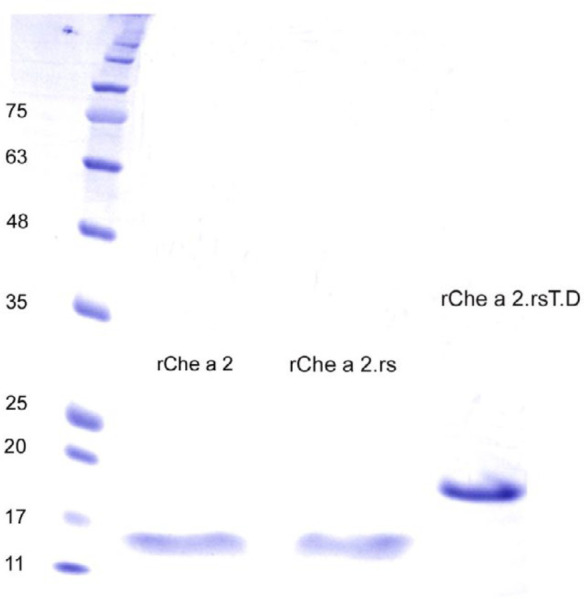
SDS-PAGE analysis of purified rChe a 2, Che a 2.rs, and Che a 2.rs^T.D^

**Figure 3:**
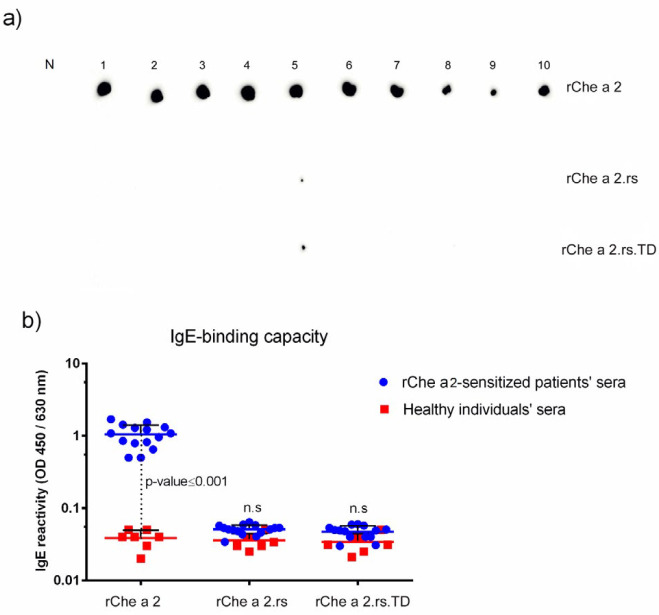
The IgE-reactivity of rChe a 2, rChe a 2.rs, and rChe a 2.rs^T.D^

The recombinant proteins were successfully expressed in *E. coli*, purified by means of metal affinity chromatography and subjected to SDS-PAGE analysis (Figure 2). As shown in Figure 3, typical protein band could be observed on the coomassie blue-stained SDS-PAGE slabs, in which the band with the molecular weight of approximately 15.6 kDa corresponds to Che a 2.rs (containing His-tag, two added amino acids by xho1 site and the initiator methionine), and the bigger protein band (approximately 19.6 kDa) corresponds to Che a 2.rs^T.D ^(containing His-tag, two added amino acids by xho1 site and the initiator methionine).

The IgE-reactivity of r.Che a 2, r.Che a 2.rs, or r.Che a 2.rs^T.D ^in patients with allergy to r.Che 2 was evaluated by dot blot and indirect ELISA (Figure 3). Dot blot results demonstrated a significant reduction in IgE-reactivity of both restructured variants including r.Che a 2.rs and r.Che a 2.rs^T.D^ for all 10 patients tested (Figure 3a). IgE-binding ELISA results with serum from 15 patients also confirmed a considerable reduction in the IgE-binding capacity of r.Che a 2.rs and r.Che a 2.rs^T.D^ compared with r.Che a 2 (Figure 3b). 

***Che a 2.rs*** ^T.D^ ***produces Che a 2-specific IgG more potently than Che a 2.rs in mice***

**Figure 4:**
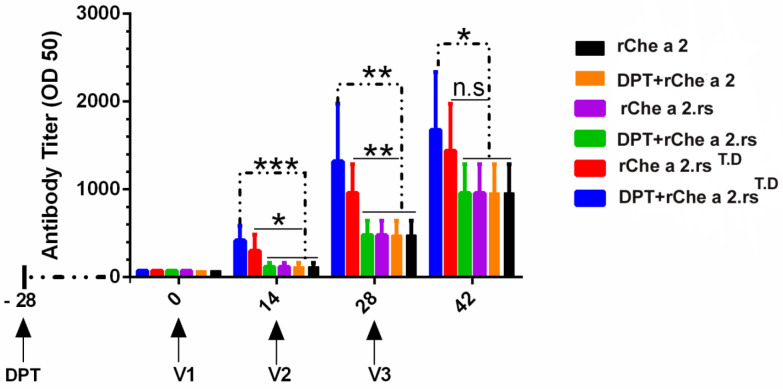
Evaluation of Che a 2-specific IgG antibody titer in young immunized mice by indirect ELISA

The production of Che a 2-specific IgG antibody was compared in mice sera after immunization with r.Che a 2, Che a 2.rs, or Che a 2.rs^T.D^, using ELISA. A high-titer of Che a 2-specific IgG antibody was observed in mice immunized with the vaccine containing universal CD4^+^ T cell epitope from tetanus and diphtheria toxoid (Che a 2.rs^T.D^) compared with those immunized with rChe a 2 or Che a 2.rs (Figure 4). Furthermore, pre-vaccination of mice with DPT vaccine-induced a rapid and significant increase of the antibody response in mice vaccinated with Che a 2.rs^T.D^.

**Figure 5:**
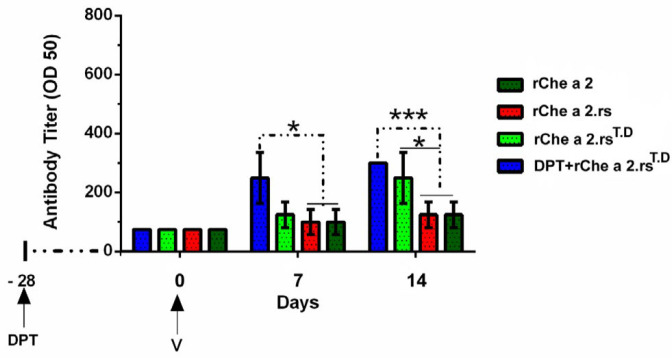
Evaluation of Che a 2-specific IgG antibody titer under limiting condition

In order to determine if the incorporation of the universal CD_4_^+^ T-Cell epitopes from tetanus and diphtheria toxoid could also enhance the antibody response under sub-optimal vaccination conditions, including poor responders (aged mice), we also immunized some old mice with very low doses of the antigen. Results indicated that the only vaccine containing the universal CD4^+^ epitopes from tetanus and diphtheria toxoid, r.Che a 2.rs^T.D^, could significantly boost Che a 2–specific IgG response in aged mice. The level of Che a 2-specific IgG antibody titer was higher in mice immunized with r.Che a 2.rs^T.D^, so that it was five times greater than those mice immunized with r.Che a 2.rs (Figure 5), and this response became 12.21 times greater in mice pre-vaccinated with the DPT vaccine, while the other vaccine elicited significantly lower antibody responses in mice.

***Incorporation of TD epitope in Che a 2.rs enhances production of IgG, capable of blocking IgE-binding***

**Figure 6:**
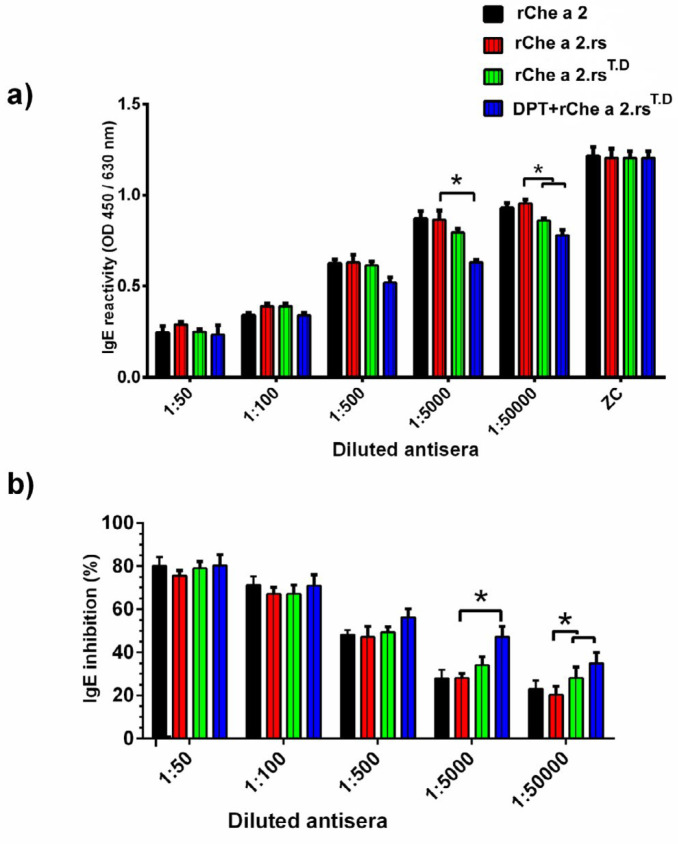
Competitive inhibition ELISA (ciELISA) for measurement of rChe a 2-specific IgE-inhibition by IgG antibody in immunized mice sera

The percentage of inhibition of IgE-binding to rChe a 2 in immunized mice sera (containing Che a 2-specific IgG) was measured with ciELISA. The results showed that sera from mice immunized with r.Che a 2.rs^T.D ^exhibited greater inhibitory activity against IgE-binding to rChe a 2 in comparison to sera from mice immunized with r.Che a 2.rs (Figures 6 a, b). Prevaccination of mice with DPT vaccine-induced higher inhibitory activity than sera from mice immunized with r.Che a 2.rs^TD ^that were not prevaccinated. The mean percentages of IgE-binding inhibition to rChe a 2 by anti-r.Che a 2.rs^T.D ^antibody from mice pre-vaccinated with DPT vaccine were 1.67- and 1.35-fold greater than sera from mice vaccinated with r.Che a 2.rs in mouse sera with 1:5000 to 1:50000 dilutions, respectively. Evaluation of blocking IgG response in old mice (10 months) immunized with low antigen dose revealed that the production of blocking IgG antibody in mice immunized with r.Che a 2.rs was very low, while r.Che a 2.rs^T.D^ enhances blocking IgG production considerably, even under sub-optimal vaccination conditions. The mean percentages of inhibition of IgE-binding to Che a 2 by sera from mice immunized with r.Che a 2.rs^T.D ^or r.Che a 2.rs were 50 % or 13 %, respectively.

***Incorporated T-cell epitopes from tetanus and diphtheria toxins are recognized by lymphocytes***

The MTT proliferation assay was used to determine if the incorporation of T-cell epitopes from tetanus and diphtheria toxoids into the protein vaccine can increase the proliferation of the lymphocytes obtained from patients with allergy to Che a 2. The incorporated T-cell epitopes from tetanus and diphtheria toxoids into Che a 2.rs^T.D ^vaccine increased the proliferation of lymphocytes more potently than r.Che a 2.rs. Evaluation of pro-allergenic cytokines from stimulated PBMC revealed that r.Che a 2.rs- and r.Che a 2.rs^T.D^-stimulated PBMCs secreted significantly lower levels of IL-5 and IL-13, compared with those stimulated with r.Che a 2.

## Discussion

**Figure 7:**
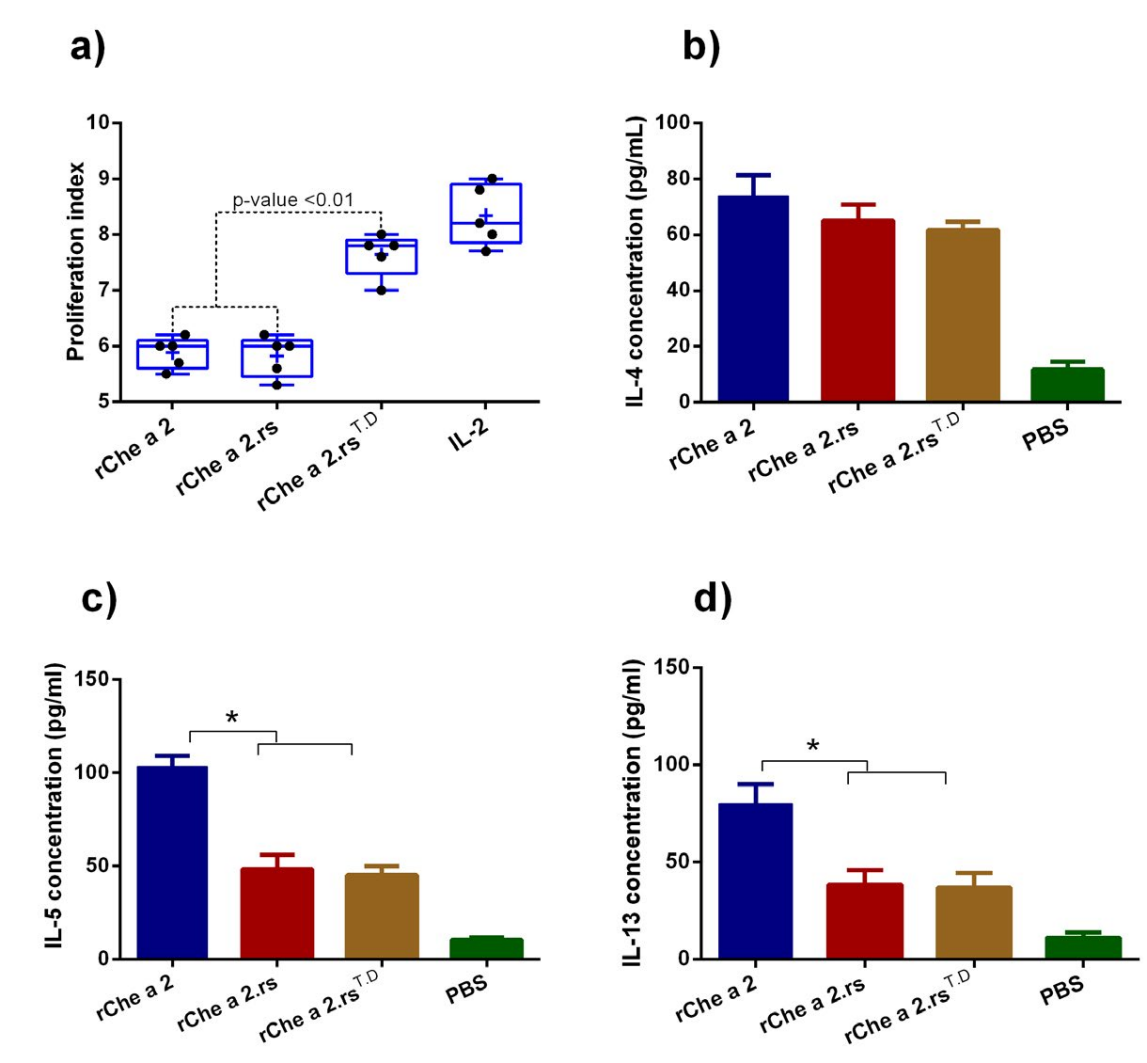
Lymphocyte proliferation and pro-allergenic cytokine induced by recombinant proteins

The current study provides evidence that incorporation of two powerful and universal T-cell epitopes from tetanus and diphtheria toxoids into a hypoallergenic vaccine could potentially improve protective allergen-specific IgG response, which may be important in allergy immunotherapy (34, 35). Disruption of linear- and conformational-type IgE epitopes either by deletion, inversion, or a mosaic restructuring are the most well-known strategies to generate hypoallergenic derivatives from allergens (19, 36). However, these strategies might destroy some T-cell epitopes and subsequently reduce vaccine-induced antibody response, as well as limit the population coverage of vaccine. It seems that the incorporation of exogenous universal T helper cell epitopes from tetanus (T830-844) and diphtheria (D331-345) may increase hypoallergen-induced IgG response, and remove problems regarding the abolishment of allergen-specific T-cell epitopes. Previous experiments demonstrated that T-cell T830-844 and D331-345 epitopes bind to various MHC class II alleles that result in the employment of CD4^+^ T helper cells involved in B-cell maturation. Also, the presence of pre-existing CD4^+^ cell memory to the mentioned TD epitopes in tetanus and diphtheria vaccinated individuals can induce a rapid and robust antibody response against the TD-epitope-fused allergen (21, 34, 35, 37, 38). In the current study, we designed a hypoallergenic variant for profilin from *C. album* (Che a 2), termed r.Che a 2.rs, in accordance with the strategy used to generate hypoallergenic Phl p 12 and profilin from timothy grass (26). Che a 2.rs exhibited significantly diminished IgE-binding compared with r.Che a 2, which is similar to what is observed with the hypoallergenic derivative of Phl p 12 generated by Westritschnig *et al.* (26), and confirms the crucial role of conformational-type IgE epitope in the allergenic activity of profilins. Incorporation of T830-844 and D331-345 epitopes in the designed hypoallergenic vaccine significantly enhanced IgG production in mice, capable of inhibiting IgE-binding from the serum of allergic patients to Che a 2. This could be due to the stimulation of allergen-specific IgG antibody-producing B-cells by TD epitopes-specific T-cells. In addition, Che a 2.rs^T.D^ dramatically induced a rapid and robust IgG response in mice prevaccinated with DPT compared with the non-prevaccinated ones. This can be the reason behind the pre-existing TD epitopes-specific T-cell memory in mice prevaccinated with DPT. In various experiments, T830-844 was fused to antigen in order to recruit T-cell ‘help’ to B-cell producing antibodies, such as MAG-Tn3 therapeutic vaccine that is under phase 1 clinical trial. MAG-Tn3 contains α-D-N-acetylgalactosamine (Tn antigen as B-cell epitope) conjugated to T830-844 epitope (35). Ahlborg *et al.* (34) demonstrated that linkage of T830-844 epitope to C-terminal region, derived from merozoite surface protein 1 (MSP119), induced protective IgG response against malaria in mice. Fraser *et al.* (21) demonstrated that a dimeric MHC class II binding peptide including T830-844 epitope and D331-345 linked with an internal cathepsin cleavage site could improve population coverage and induce greater IgG response compared with individual peptides. The T.D epitopes induced a memory recall in all PBMC obtained from humans, and conjugation of TD epitope to nicotine nanoparticles produced rapid and robust anti-nicotine antibody. The immunogenicity assessment of Che a 2.rs^T.D ^and Che a 2.rs in suboptimal immunization conditions, including aged mice immunized with low-dose vaccine revealed that TD epitopes could enhance the immunogenicity of the vaccine even in poor responders. According to the increasing rates of allergies in elderly people (39, 40), and decreasing naive T-cells and naive B-cells with aging, fusion of a universal TD epitope in hypoallergenic vaccines may be a potential method to enhance immunogenicity of hypoallergenic vaccines in elderly patients by recruiting more T-cell and pre-existing T-cell in individuals that received the DPT vaccine. As shown in Figure 7, we found that lymphocytes stimulated with the Che a 2rsTD show comparatively high capacity in the proliferation of lymphocytes obtained from patients with allergy and a decrease in allergy-inducing cytokine secretion.

a) The ribbon representation of the structure of Che a 2 predicted by Swiss model. b) Linear representation of wild-type Che a 2, restructured Che a 2 (Che a 2.rs) and Che a 2.rs fused from C-terminus to T-cell epitopes from tetanus and diphtheria toxoid (Che a 2.rs^T.D^). CS represents the cathepsin cleavage site (PMGLP)

**Table1:** Demographic and serological data of Chenopodium album-allergic patients

Patients	Age (years)	Gender	*C. album* Specific IgE (IU/ml)	Symptoms
1	24	M	8.4	As, Rc
2	32	M	9.8	Rc
3	18	F	7.4	R
4	25	M	8.3	RC
5	38	F	9.4	R
6	32	F	6.2	R, AS
7	40	F	8.2	RC
8	35	M	4.5	R
9	26	M	7.8	R
10	37	M	6.6	Rc, As
11	23	F	8.4	Rc
12	21	M	7.3	Rc
13	36	F	8.5	Rc
14	27	M	7.9	R
15	31	M	7.4	R

Rc: rhinoconjunctivitis; R: rhinitis; As: Asthma; M: male; F: female

Expressed proteins rChe a 2 (15.6 kDa), rChe a 2.rs (15.6 kDa), and rChe a 2.rs_T.D_ (19.5 kDa) were loaded on 15% polyacrylamide SDS-PAGE gel and their molecular weights were evaluated using low molecular weight protein marker. Symbols: Che a 2.rs, restructured Che a 2; Che a 2.rs^T.D^, restructured Che a 2 fused to CD4 epitope of diphtheria and tetanus toxoid; rChe a 2, recombinant Che a 2

a) Disk-blotted rChe a 2, rChe a 2.rs, and rChe a 2.rs^T.D^ were evaluated by Che 2-sensitive patients’ sera (n=10). Serum of only one patient (No.5) showed very low IgE-reactivity with restructured variants of Che a2 (rChe a 2.rs and rChe a 2.rs^T.D^), too. b) Comparison of IgE-reactivity of rChe a 2, rChe a 2.rs, and rChe a 2.rs^T.D^ with sera from rChe a 2-sensitive patients (n=15) and healthy individuals (n=7). Data shows the mean+SD of triplicate wells per group

BALB/c mice (8 weeks) were pre-vaccinated with DPT vaccine or left unvaccinated. After 3 weeks, mice (five mice per group) were immunized three times (at 0, 14, 28 days) with rChe a 2, rChe a 2.rs, or rChe a 2.rs^T.D^ and the level specific IgG antibody titer against recombinant Che a 2 (rChe a 2) were evaluated for each time point. A significance value (*P-value*) determined between antibody titer in mice immunized with rChe a 2.rs^T.D ^(prevaccinated with DPT vaccine or without prevaccination) and Che a 2.rs. Symbols: *, *P-value ≤*0.05; **, *P-value ≤*0.01;***, *P-value ≤*0.005

Four groups of nine-month-old female BALB/c mice (five mice per group) were pre-vaccinated with the DPT vaccine or left unvaccinated, after 4 weeks, mice were immunized with sub-optimal dose (1 μg/ml) of rChe a 2.rs or rChe a 2.rs^T.D^, and immunogenicity of antigens was assessed at 0, 7, and 14 days after vaccination. A significant difference was found between the titer of the antibody in mice immunized with Che a 2.rsT.D (primed with DPT vaccine or not primed) and Che a 2.rs. Symbols: *, *P-value ≤*0.05; **, *P-value ≤*0.01;***, *P-value ≤*0.005

a) ELISA wells coated with rChe a 2 were incubated with a mixture of sera composed of 1:5-diluted pooled sera from rChe a 2-sensitive patients (n=10) and various dilutions of sera (1:50, 1:100, 1:500, 1:5000, and 1:50000) from immunized mice (n=5). IgE-binding capacity of rChe a 2 was measured at 450/630 nm. b) The figure shows the percentage of inhibition of IgE-binding to rChe a 2 by various dilutions of anti-rChe a 2, anti-rChe a 2.rs, or anti-rChe a 2.rs^T.D^ antibodies. * represents *P-value<*0.05

a) PBMC from Che a 2-sensitized patients (n=5) were stimulated with rChe a 2, rChe a 2.rs, rChe a 2.rs^T.D^, or IL2 (as positive control) and lymphocyte proliferation was measured and expressed as lymphocyte proliferation index (y-axis). Plus sign (+) represents the mean data. b-d) Comparison of the pro-allergenic cytokines (IL4, IL5, IL13) secreted by PBMC of rChe a 2-sensitized patients (n=5) stimulated by rChe a 2, rChe a 2.rs, rChe a 2.rs^T.D^, or PBS as negative control. * represents *P-value<*0.05

## Conclusion

Development of allergen-specific IgG is one of the purposes and signs of successful specific immunotherapy. The current study demonstrates that incorporation of powerful and universal epitopes from tetanus and diphtheria toxoids in a hypoallergenic variant of an allergen vaccine could improve protective antibody response, even under suboptimal immunization condition. This study also provides an efficient strategy for generation of blocking antibody responses against allergens, even in suboptimal immunization conditions.
